# Aflatoxin Exposure and Viral Hepatitis in the Etiology of Liver Cirrhosis in The Gambia, West Africa

**DOI:** 10.1289/ehp.11661

**Published:** 2008-07-10

**Authors:** Mark H. Kuniholm, Olufunmilayo A. Lesi, Maimuna Mendy, Aliu O. Akano, Omar Sam, Andrew J. Hall, Hilton Whittle, Ebrima Bah, James J. Goedert, Pierre Hainaut, Gregory D. Kirk

**Affiliations:** 1 Infectious Disease Program, Department of Epidemiology, Johns Hopkins Bloomberg School of Public Health, Baltimore, Maryland, USA; 2 International Agency for Research on Cancer, Gambia Hepatitis Intervention Study, Banjul, The Gambia; 3 Department of Medicine, Lagos University Teaching Hospital, Lagos, Nigeria; 4 Medical Research Council Laboratories, Banjul, The Gambia; 5 Department of Medical Services, Royal Victoria Teaching Hospital, Government of The Gambia, Banjul, The Gambia; 6 Department of Radiodiagnosis, National Hospital, Abuja, Nigeria; 7 London School of Hygiene and Tropical Medicine, London, United Kingdom; 8 Infections and Immunoepidemiology Branch, Division of Cancer Epidemiology and Genetics, Department of Health and Human Services, National Cancer Institute, National Institutes of Health, Department of Health and Human Services, Bethesda, Maryland, USA; 9 International Agency for Research on Cancer, Gambia Hepatitis Intervention Study, Lyon, France

**Keywords:** aflatoxin, Africa, hepatitis B virus, liver cirrhosis, p53, The Gambia, ultrasound

## Abstract

**Background:**

Cirrhosis of the liver is thought to be a major cause of morbidity and mortality in sub-Saharan Africa, but few controlled studies on the etiology of cirrhosis have been conducted in this region.

**Objectives:**

We aimed to elucidate the association between environmental and infectious exposures and cirrhosis in The Gambia.

**Methods:**

Ninety-seven individuals were diagnosed with cirrhosis using a validated ultrasound scoring system and were compared with 397 controls. Participants reported demographic and food frequency information. Blood samples were tested for hepatitis B surface antigen (HBsAg), hepatitis B e antigen (HBeAg), hepatitis C virus (HCV) antibody, HCV RNA, and the aflatoxin-associated 249^ser^ TP53 mutation.

**Results:**

HBsAg seropositivity was associated with a significant increase in risk of cirrhosis [odds ratio (OR) = 8.0; 95% confidence interval (CI), 4.4–14.7] as was the presence of HBeAg (OR = 10.3; 95% CI, 2.0–53.9) and HCV infection (OR = 3.3; 95% CI, 1.2–9.5). We present novel data that exposure to aflatoxin, as assessed both by high lifetime groundnut (peanut) intake and by the presence of the 249^ser^ TP53 mutation in plasma, is associated with a significant increase in the risk for cirrhosis (OR = 2.8; 95% CI, 1.1–7.7 and OR = 3.8; 95% CI, 1.5–9.6, respectively). Additionally, aflatoxin and hepatitis B virus exposure appeared to interact synergistically to substantially increase the risk of cirrhosis, although this was not statistically significant.

**Conclusions:**

Our results suggest that the spectrum of morbidity associated with aflatoxin exposure could include cirrhosis.

Hepatocellular carcinoma (HCC) is a leading cause of cancer death worldwide [World Health Organization (WHO) 2003], and a heavy burden of HCC has been documented in sub-Saharan Africa (Bah et al. 2001; Bosch et al. 2005). Chronic infection with hepatitis B virus (HBV) is endemic in sub-Saharan Africa, and hepatitis C virus (HCV) infection is also present (McMahon 2005; The Global Burden of Hepatitis C Working Group 2004). Dietary exposure to aflatoxin, primarily through ingestion of contaminated maize and groundnuts (peanuts), is also widespread (Turner et al. 2005; Wild et al. 1993). Largely because of the lack of clinical and research infrastructure, rigorous investigation into the etiology and characteristics of chronic liver disease in sub-Saharan Africa has been limited. Studies on HCC are relatively rare, and controlled studies on the etiology of cirrhosis have been reported even less frequently (Lesi et al. 2002).

Worldwide, cirrhosis of the liver is the 16th leading cause of death, responsible for hundreds of thousands of deaths each year (WHO 2003). Cirrhosis onset is often asymptomatic or associated with mild clinical symptoms, and individuals with subclinical cirrhosis can lead relatively normal lives for many years. Cirrhotic persons, however, are at high risk for liver decompensation and, irrespective of etiology, have a high risk for development of HCC. Diagnosis of cirrhosis, generally requiring histopathologic review of a liver biopsy specimen, is infrequently performed in many resource-constrained settings. In developed countries, cirrhosis is associated with chronic infection with HBV and HCV viruses (Corrao et al. 1998; Tsai et al. 1994, 2003), excessive use of alcohol (Corrao et al. 1998; Tsai et al. 2003), hereditary factors (Gershwin et al. 2005), obesity (Poonawala et al. 2000), smoking (Tsai et al. 2003), and occupational exposure to vinyl chloride (Mastrangelo et al. 2004), but evaluation of potential interactions between these risk factors are only beginning to be conducted (Corrao et al. 1998; Mastrangelo et al. 2004). Additionally, it is not known whether recognized etiologic factors for cirrhosis constitute an exhaustive list or whether unidentified etiologic agents remain.

HCC most commonly occurs in cirrhotic individuals (Chen et al. 2006; Takano et al. 1995). Risk factors for HCC have been extensively studied, and in addition to the etiologic factors for cirrhosis listed above, HCC is also associated with aflatoxin exposure [International Agency for Research on Cancer (IARC) 1993; Qian et al. 1994]. Although the biologic mechanisms that induce cirrhosis and carcinogenesis are different, the considerable overlap in causative factors between cirrhosis and HCC suggests that factors that have thus far only been linked to HCC may also predispose to cirrhosis.

In this study, we investigated the diagnosis of liver cirrhosis via a noninvasive, validated ultrasound scoring system (Hung et al. 2003; Lin et al. 1993) among individuals with suspected liver disease in The Gambia, West Africa. Then, in a case–control study design, we examined environmental exposures for cirrhosis, including viral, dietary, and lifestyle risk factors in this population. Finally, we explored interactions between HBV and aflatoxin exposure to more precisely describe the etiology of this major public health problem in a sub-Saharan African population.

## Materials and Methods

### Study population

The study population was recruited as a part of the Gambia Liver Cancer Study as described previously (Kirk et al. 2004). Briefly, individuals with suspected liver disease were recruited from the liver disease referral clinics of three tertiary hospitals in The Gambia [Royal Victoria Hospital (RVH), Banjul; Medical Research Council Hospital (MRC), Fajara; and Bansang Hospital (BSG), Bansang]. Recruitment occurred between September 1997 and January 2001. After informed consent, study participants with suspected liver disease were administered a questionnaire by an interviewer, underwent standardized clinical and ultrasound exams, and provided a blood sample. The questionnaire assessed demographic, medical history, food consumption, and lifestyle characteristics. All questionnaire, clinical, and ultrasound information was recorded on standardized reporting forms. All ultrasound examinations were performed by physicians trained in liver ultrasonography; periodic retraining and selected duplicate testing by examiners were performed to ensure consistency.

Individuals without suspected liver disease were recruited from the outpatient clinics of the same three hospital sites to serve as a control group. These participants were frequency-matched to suspected liver disease patients by age (10-year groupings) and sex. Participants without suspected liver disease were administered the identical interviewer-administered questionnaire, underwent a standardized clinical examination to identify signs of underlying liver disease, and provided a blood sample. Local and international scientific and ethical review committees approved the study protocol, and informed consent was obtained from each participant before inclusion in the study.

### Case and control definitions

Individuals with suspected liver disease recruited from the liver disease referral clinics were classified as having liver cirrhosis (cases) if they had *a*) no evidence of space-occupying hepatic lesions by ultrasound examination; *b*) no evidence for alternative diagnoses (e.g., congestive heart failure, abdominal tuberculosis); *c*) complete demographic and hepatitis B surface antigen (HBsAg) information; and *d*) a score of 7 of a possible 11 points on the ultrasound-based cirrhosis scale developed by Lin et al. (1993). This scale has a 77.8% sensitivity and 92.5% specificity versus liver biopsy to classify HBV-infected patients as having cirrhosis (Hung et al. 2003) and is increasingly used as a non-invasive mechanism to diagnose cirrhosis (Iloeje et al. 2006). Individuals without suspected liver disease recruited from the out-patient clinics were classified as controls if they had *a*) alpha-fetoprotein (AFP) levels in the normal range (< 20 ng/mL); *b*) no evidence of underlying liver disease on the standardized clinical exam; and *c*) complete demographic and HBsAg information.

### Laboratory testing

Blood specimens were processed promptly after collection and stored at either –20°C or –70°C, depending on the testing planned for each aliquot. HBsAg was determined as a marker of chronic HBV carriage by a reverse passive hemagglutination assay (Murex Diagnostics Limited, Dartford, UK) with radioimmunoassay testing of negative samples (Sorin Biomedica Diagnostics, Vercelli, Italy). Participants positive for HBsAg were tested for hepatitis B e antigen (HBeAg) using a radioimmunoassay kit (DiaSorin SA, Sallugia, Italy). HCV antibody status (anti-HCV) was determined by third-generation enzyme-linked immunosorbent assay (ELISA; ORTHO Clinical Diagnostics, Neckargemund, Germany). Samples that were seroreactive by ELISA to HCV were screened with a recombinant immunoblot assay (RIBA HCV 3.0 SIA; Chiron, Emeryville, CA, USA) and for HCV RNA using a real-time reverse transcription-polymerase chain reaction (RT-PCR) assay as described previously (Zhang et al. 2006). We defined individuals who tested positive on either the RIBA assay or the RT-PCR assay as being HCV-positive. AFP was detected and quantified by standard radiometric assay methods (DiaSorin).

### Detection of the 249^ser^ TP53 mutation

Previous investigations have demonstrated that the 249^ser^ TP53 mutation is a biomarker of the aflatoxin-associated mutational effect in the *p53* gene (Aguilar et al. 1993; Mace et al. 1997); methods for detection of the 249^ser^ TP53 mutation in plasma have been previously described (Kirk et al. 2000, 2005; Szymanska et al. 2004). Briefly, circulating cell-free DNA was extracted from 200 μL plasma using QiAmp DNA Blood Mini Kit (Qiagen, Hilden, Germany) according to the manufacturer’s protocol. Purified DNA was eluted and resuspended, and exon 7 of the *TP53* gene was amplified. PCR products underwent restriction digestion (*Hae*III) followed by automated sequencing (AbiPrism 3100; PerkinElmer, Waltham, MA, USA). All samples underwent two independent analyses, and discordant results underwent a third analysis. At all steps, a series of controls without DNA template was used to rule out contamination.

### Statistical analysis

We calculated descriptive statistics for questionnaire and laboratory variables among case and control participants using *t*-tests, asymptotic chi-square tests, and Fisher’s exact tests to test for differences between study groups. Logistic regression models were used to evaluate potential risk factors for cirrhosis in cases compared with controls. To minimize any potential effect of including occult HCC within our cirrhotic cases, we performed separate analyses excluding cirrhosis cases with significantly elevated AFP levels (> 500 ng/mL); however, our findings were not substantively changed (data not shown). We evaluated markers of viral hepatitis infection alone and with adjustment for age, sex, recruitment site and date, socioeconomic status (e.g., education, household floor type), smoking, alcohol consumption, and other viral markers.

Dietary and molecular markers of aflatoxin exposure, both alone and with adjustment for potential confounding factors, were evaluated as possible risk factors for cirrhosis. Self-reported dietary exposure to aflatoxin was constructed from the dietary questionnaire. Study participants were asked about their consumption of groundnuts in the past 2 months and whether this consumption was greater, less, or about the same as during the entirety of their lives. Previously, we demonstrated that persons with clinical liver disease often decrease their dietary intake as well as reduce exposure to risk factors suspected to be associated with the disease (Kirk et al. 2005); therefore, lifetime rather than 2-month groundnut intake was considered the primary dietary exposure variable. Lifetime groundnut consumption was determined by adjusting the level of 2-month groundnut consumption up one level of consumption, or down one level of consumption for those individuals who reported decreased or increased lifetime consumption relative to recent intake, respectively. We used this variable for trend analysis, but collapsed this variable into three levels of exposure in our evaluations of risk. Similar to the dietary exposure variables, short-term bio-markers of aflatoxin exposure such as urinary adducts or serum aflatoxin–albumin adducts can be affected by the severity of liver disease in a case–control study design (Hall and Wild 1994). Therefore, we evaluated plasma 249^ser^ TP53 mutations, which, although perhaps less sensitive, would be reflective of the biological effects of longer-term aflatoxin exposure and be less susceptible to changes with illness. The plasma 249^ser^ TP53 mutation was detected more often among individuals with higher levels of lifetime groundnut consumption, but this association did not attain statistical significance (exact *p* = 0.10). Therefore, although related, the dietary and molecular markers represent different measures of aflatoxin exposure; we used separate models for analysis of each aflatoxin marker.

We evaluated interactions between aflatoxin exposure and HBV infection through participant stratification and subsequent logistic regression analysis, as well as through incorporation of first-order interaction terms into the logistic regression models. All analyses were conducted using SAS version 9.1 (SAS Institute, Inc., Cary, NC, USA).

## Results

### Characteristics of the study participants

After excluding liver-disease referral patients with space-occupying lesions on ultrasonography characteristic of HCC or with alternative diagnoses other than liver disease (Kirk et al. 2004), a total of 111 persons with suspected liver cirrhosis were identified. Of these, two were not tested for HBsAg. Of the remaining 109 individuals, 97 had a score of ≥ 7 on the ultrasound-based cirrhosis scale of Lin et al. (1993) and were classified as cirrhosis cases. Of 408 control participants recruited, 397 had no evidence of underlying liver disease, AFP levels in the normal range, and complete HBsAg and demographic information and were included in this analysis.

Characteristics of the 97 cirrhosis cases and 397 controls are listed in Table 1. Of the case and control participants, mostly male, they often lived in homes with dirt floors, and they were recruited throughout the year and from all adult age brackets. The mean age of cirrhotic patients was 42.5 years compared with 44.8 years for controls. Cirrhotic patients differed from control participants with regard to recruitment site and ethnicity and were less likely to have attended school.

### HBV and HCV infection and cirrhosis risk

Table 2 shows markers of HBV and HCV infection among cirrhosis cases and controls, as well as unadjusted and adjusted odds ratios (ORs) for cirrhosis. HBsAg seropositivity was very common among the cirrhosis cases (58.8%) and highly associated with cirrhosis [adjusted OR = 8.0; 95% confidence interval (CI), 4.4–14.7]. Individuals who were HBeAg-positive had a 10-fold higher risk of cirrhosis than persons without chronic HBV infection. HCV infection was identified in 9.3% of cirrhotic cases and was associated with a > 3-fold higher risk of cirrhosis compared with HCV-uninfected persons.

### Aflatoxin exposure and cirrhosis risk

Eighty cirrhosis cases and 327 controls provided information regarding groundnut consumption on the study questionnaire. In addition, 78 cirrhosis cases and 346 controls were evaluated for plasma 249^ser^ TP53 mutations. Table 3 shows the distribution of lifetime groundnut consumption and of 249^ser^ TP53 mutations among cirrhosis cases and controls, as well as unadjusted and adjusted ORs evaluating the association between aflatoxin exposure variables and cirrhosis. Increasing lifetime groundnut intake was associated with a significant increase in the risk of cirrhosis (*p*-trend = 0.01). Moderate and high lifetime groundnut consumption versus low consumption was associated with an almost 2-fold and significant 3-fold increase in risk of cirrhosis, respectively. The presence of the plasma 249^ser^ TP53 mutation conferred a statistically significant almost 4-fold increase in risk of cirrhosis in this population.

### Interaction between HBV infection and aflatoxin exposure

Table 4 shows the separate and joint distribution of HBV and aflatoxin markers among cirrhosis cases and controls, as well as adjusted ORs evaluating the association with cirrhosis. Compared to persons with low-to-moderate groundnut intake that were HBsAg-negative, the risk of cirrhosis was increased 1.7-fold with high groundnut intake alone, > 8-fold with HBsAg-positivity alone, and almost 27-fold in individuals with both exposures present. As seen in Table 4, the risk of cirrhosis increased almost 2-fold with expression of the 249^ser^ TP53 mutation alone and > 7-fold with HBsAg positivity, but was 46-fold higher for persons expressing both compared with individuals who lacked either marker. The statistical term for the interaction between HBsAg and either lifetime groundnut intake or the 249^ser^ TP53 mutation variables did not attain significance (*p* = 0.37 and 0.35, respectively).

## Discussion

Despite the postulated heavy burden of disease, remarkably little descriptive risk factor or natural history data have been published on chronic liver disease or cirrhosis from sub-Saharan African populations (Lesi et al. 2002; Lin et al. 2005). In this study, we found that chronic HBV infection and aflatoxin exposure, either separately or in synergy, were the etiologic agents likely responsible for most cirrhosis cases identified in this West African study population. Our results suggest that the spectrum of morbidity associated with aflatoxin exposure could include cirrhosis.

The impact of aflatoxin on human health is substantial and has been documented in both acute and chronic exposure settings (Groopman et al. 2008; Strosnider et al. 2006). Acute exposure to high levels of aflatoxin (aflatoxicosis) can result in acute toxicity, which often presents clinically as fulminant liver failure. For example, a recent outbreak of acute aflatoxicosis from contaminated maize in Kenya resulted in 125 human deaths, significant mortality among domesticated livestock, and widespread socioeconomic impact (Lewis et al. 2005). The health impacts of chronic aflatoxin exposure are equally serious. A large body of experimental, clinical, and epidemiologic evidence has defined aflatoxin as one of the most potent naturally occurring hepatocarcinogens; chronic exposure to moderate or even low levels of aflatoxin has been linked to development of HCC (IARC 1993). Chronic aflatoxin exposure has also been associated with impaired growth and perturbations in measures of immune function in young West African children (Gong et al. 2004; Turner et al. 2003). However, data regarding other potential health effects of chronic aflatoxin exposure are scarce, resulting in significant limitation of current research (Strosnider et al. 2006). Primary limitations for conducting this research include difficulties in defining clinical outcomes in often remote or resource-constrained environments and difficulty in accurately assessing aflatoxin exposure.

Although standardized clinical scoring systems, such as the Child-Pugh score, have for decades been accepted for assessing the severity of liver cirrhosis, numerous recent reports have increasingly focused on the utility of noninvasive methods and clinical prediction models to diagnose fibrosis and cirrhosis (Schneider et al. 2005; Shen et al. 2006; Yamada et al. 2006). However, most of these studies to date have been conducted among HCV-infected populations in industrialized countries. In the present study we used a validated, reproducible, and non-invasive diagnostic method to diagnose cirrhosis in a setting where an etiologic study of risk factors for biopsy-diagnosed liver cirrhosis would not have been possible. The presence of encephalopathy or marked ascites precluded biopsy in some study participants, and resources for rapidly performing pre-biopsy screening laboratories were not consistently available at the three recruitment sites. Furthermore, participant acceptance of ultrasound was excellent, and potentially serious postbiopsy complications were summarily avoided. Last, given that the specificity of the ultrasound scoring system used in this study is > 90% among HBV-infected subjects (Hung et al. 2003), we believe that noninvasive methods for measuring liver fibrosis and diagnosing cirrhosis hold significant promise for increasing clinical and research capabilities in African settings.

Currently there is no gold standard methodology available to measure cumulative lifetime aflatoxin exposure. In lieu of a gold standard, we used two different approaches to assess exposure to aflatoxin during the period of time that we hypothesized would have etiologic significance for the development of cirrhosis. First, we evaluated self-reported lifetime intake of groundnuts, the primary food component contributing to aflatoxin exposure in The Gambia (Wild et al. 1993, 2000). Questionnaire-derived data have been validated for assessment of many dietary components; however, this information is obviously dependent on participant recall for its validity. Second, we evaluated a laboratory-based bio-marker potentially reflecting the biological effect of aflatoxin exposure. The presence of the 249^ser^ TP53 mutation, associated with aflatoxin exposure in experimental systems (Aguilar et al. 1993; Mace et al. 1997), is commonly observed in HCC patients from high aflatoxin exposure regions (Aguilar et al. 1994) and is strongly associated with HCC in case–control and prospective epidemiologic studies (Jackson et al. 2003; Kirk et al. 2000, 2005). However, recent experimental data suggest that aflatoxin exposure alone may not be sufficient to induce 249^ser^ TP53 mutations (Tong et al. 2006) and that other cofactors such as HBV infection (Sohn et al. 2000) or host factors (Mace et al. 1997) may modulate the mutagenic capacity of aflatoxin. In addition, because detection occurs in plasma DNA, it is unclear if advanced liver disease impacts release of the mutation. Finally, levels of the 249^ser^ TP53 mutation in plasma exhibit seasonal variability (Lleonart et al. 2005), suggesting that this marker may partly reflect shorter-term effects of aflatoxin exposure. Longitudinal studies to determine marker stability over time and the effect of liver disease severity on detection are needed; these issues are the subject of ongoing investigations in The Gambia. Despite these limitations, we believe that the 249^ser^ TP53 mutation represents the best biomarker currently available to assess the biological effect of cumulative aflatoxin exposure.

Both measures of aflatoxin exposure evaluated as a part of this study were associated with a significant increase in the risk of cirrhosis. Our results also suggest that aflatoxin exposure and chronic HBV infection may interact synergistically to increase the risk of cirrhosis notably above and beyond that expected for two independent hepatotoxins. The mechanisms by which aflatoxin is activated and induces HCC-associated mutational changes have been well characterized (Wild and Turner 2002), but less well understood is the impact of aflatoxin on the development of cirrhosis and precancerous architectural changes in the liver. Empirical evidence of AFB1-induced fibrosis and cirrhosis in humans has thus far been limited (Aguilar et al. 1994), but animal studies have found significantly more liver fibrosis among animals experimentally inoculated with aflatoxin than uninoculated control animals (Ortatatli et al. 2005; Seffner et al. 1997). In addition, although significant progress has been made with regard to understanding the mechanisms of interaction between aflatoxin and HBV in hepatocarcinogenesis (Kew 2003), it is not clear whether these proposed mechanisms will explain the interaction between aflatoxin and HBV in the etiology of cirrhosis.

As expected, we found HBsAg positivity to be a significant risk factor for cirrhosis; we have extended this work to document a further increase in risk of cirrhosis associated with HBeAg seropositivity. Similarly, we found HCV infection to be a significant risk factor for cirrhosis, although the magnitude of this risk was lower than that conferred by HBsAg positivity. Notably, the average age of HCV-related cirrhosis patients was around 15 years older than HBV-related patients, consistent with the premise of a different natural history of infection between the two viruses in West Africa (for further discussion, see Kirk et al. 2004, 2006). In contrast, we observed no significant associations between alcohol and tobacco exposure and cirrhosis (data not shown). The low overall prevalence and limited societal acceptance of alcohol consumption in this majority Muslim country may offer some explanation for these findings. Also, given that a significant association between tobacco exposure and cirrhosis has been only rarely observed to our knowledge (Tsai et al. 2003), it is possible that exposure to tobacco use conveys no increase or only a slight increase in risk of cirrhosis.

Cirrhosis of the liver is a major cause of morbidity and mortality in sub-Saharan Africa. Using a validated ultrasound scoring system to diagnose cirrhosis, we confirmed associations with chronic HBV and HCV infections and further provide evidence that aflatoxin may also be an etiologic factor for cirrhosis. As such, expanded access to hepatitis B vaccination programs and continued development of aflatoxin-reduction intervention programs (Groopman et al. 2008; Strosnider et al. 2006; Turner et al. 2005) could substantially decrease the burden of chronic liver disease in this region.

## Figures and Tables

**Table 1:** Characteristics of the study participants (controls, *n* = 397; cirrhosis cases, *n* = 97).

Characteristic	Controls [no. (%)]	Cirrhosis cases [no. (%)]
Sex		
Male	282 (71.0)	61 (62.9)
Female	115 (29.0)	36 (37.1)
Site*		
RVH	107 (27.0)	51 (52.6)
MRC	102 (25.7)	21 (21.7)
BSG	188 (47.4)	25 (25.8)
Age group (years)		
< 35	125 (31.5)	31 (32.0)
35–44	76 (19.1)	21 (21.7)
45–54	72 (18.1)	24 (24.7)
55–64	76 (19.1)	15 (15.5)
≥ 65	48 (12.1)	6 (6.2)
Recruitment timing		
November–January	98 (24.7)	30 (30.9)
February–April	88 (22.2)	23 (23.7)
May–July	84 (21.2)	26 (26.8)
August–October	127 (32.0)	18 (18.6)
Ethnic group*		
Mandinka	130 (32.8)	24 (24.8)
Fula	82 (20.7)	31 (32.0)
Wollof	60 (15.1)	23 (23.7)
Other	125 (31.5)	19 (19.6)
Education*		
Ever school	353 (88.9)	77 (79.4)
None	44 (11.1)	20 (20.6)
Earth floor in residence		
Yes	197 (49.6)	59 (60.8)
No	200 (50.4)	38 (39.3)
Regular tobacco use		
Cigarettes	163 (41.1)	36 (37.1)
Pipe	25 (6.3)	7 (7.2)
Chewing/snuff	18 (4.3)	8 (8.3)
Regular alcohol use	33 (8.3)	8 (8.3)
Age [years (mean ± SD)]	44.8 ± 15.2	42.5 ± 14.1
Cigarette pack-years (mean ± SD)	7.4 ± 16.2	5.5 ± 12.0

* *p* < 0.05 compared with controls in that location or group.

**Table 2:** HBV and HCV infection and association with cirrhosis (controls, *n* = 397; cirrhosis cases, *n* = 97).

	Controls [no. (%)]	Cirrhosis cases [no. (%)]	Unadjusted [OR (95% CI)]	Adjusted^a^ [OR (95% CI)]
HBV status				
HBsAg (–)	336 (84.6)	40 (41.2)	Referent	Referent
HBsAg (+)	61 (15.4)	57 (58.8)	7.8 (4.8–12.8)	8.0 (4.4–14.7)
HBsAg (+)/HBeAg (+)	2 (0.5)	15 (15.5)	36.1 (8.1–161.0)	10.3 (2.0–53.9)
HCV status				
HCV (–)	381 (96.0)	88 (90.7)	Referent	Referent
HCV (+)	16 (4.0)	9 (9.3)	2.4 (1.0–5.7)	3.3 (1.2–9.5)

a Adjusted for age, sex, recruitment site and date, education, household floor type, tobacco, alcohol, HBV, and HCV variables.

**Table 3:** Exposure to aflatoxin and association with cirrhosis.

	Controls [no. (%)] *n* = 327	Cirrhosis cases [no. (%)] *n* = 80	Unadjusted [OR (95% CI)]	Adjusted^a^ [OR (95% CI)]
Lifetime groundnut intake				
Low	68 (21)	10 (13)	Referent	Referent
Moderate	189 (58)	43 (54)	1.6 (0.7–3.3)	1.7 (0.7–4.2)
High	70 (21)	27 (34)	2.6 (1.2–5.8)	2.8 (1.1–7.7)
249^ser^ TP53 mutation	*n* = 346	*n* = 78		
Absent	329 (95)	65 (83)	Referent	Referent
Present	17 (5)	13 (17)	3.9 (1.8–8.4)	3.8 (1.5–9.6)

a Adjusted for age, sex, recruitment site and date, education, household floor type, alcohol, tobacco, HBV, and HCV variables.

**Table 4:** Joint effect of HBV infection and aflatoxin exposure and association with cirrhosis.

	Controls (*n* = 327) [no. (%)]	Cirrhosis cases [no. (%)]	Adjusted^a^ [OR (95% CI)]
HBV and lifetime groundnut intake status	*n* = 327	*n* = 80	
HBsAg (–)/High intake (–)	218 (67)	25 (31)	Referent
HBsAg (–)/High intake (+)	62 (19)	12 (15)	1.7 (0.7–4.1)
HBsAg (+)/High intake (–)	39 (12)	28 (35)	8.1 (3.9–17.1)
HBsAg (+)/High intake (+)	8 (2)	15 (19)	26.8 (8.7–82.1)
HBV and 249^ser^ TP53 status	*n* = 346	*n* = 78	
HBsAg (–)/249^ser^ TP53 (–)	284 (82)	29 (37)	Referent
HBsAg (–)/249^ser^ TP53 (+)	15 (4)	4 (5)	1.8 (0.5–6.7)
HBsAg (+)/249^ser^ TP53 (–)	45 (13)	36 (46)	7.3 (3.9–13.6)
HBsAg (+)/249^ser^ TP53 (+)	2 (0.6)	9 (12)	46.0 (8.5–249.1)

a Adjusted for age, sex, recruitment site and date, education, household floor type, alcohol, tobacco, and HCV variables.
